# Orbital myeloid sarcoma misdiagnosed for subperiostal hematoma: a case report

**DOI:** 10.1186/s13256-021-03025-8

**Published:** 2021-10-08

**Authors:** Bahaa Razem, Mohamed Raiteb, Sanaa El Mrini, Faiçal Slimani

**Affiliations:** 1 Service de Stomatologie et de Chirurgie Maxillo-faciale, Hôpital 20 Août, CHU Ibn Rochd, B.P 2698, Casablanca, Maroc; 2 Faculté de Médecine et Pharmacie, Hassan II University of Casablanca, B.P 5696, Casablanca, Maroc

**Keywords:** Myeloid sarcoma, Orbit, Subperiostal hematoma, Acute myeloid leukemia

## Abstract

**Background:**

Myeloid sarcoma is a solid tumor that consists of immature myeloid cells occurring at an extramedullary site. It can present before, concurrent with, or after the diagnosis of acute myeloid leukemia or other myeloproliferative diseases, and a proportion of patients never develop bone marrow infiltration. Only a few isolated cases of pediatric orbital myeloid sarcoma have been reported, and they are often associated with a high misdiagnosis rate.

**Case report:**

We report a rare case of pediatric orbital myeloid sarcoma associated with blunt trauma in a 3-year-old Caucasian male patient, which was clinically and radiologically misdiagnosed for orbital subperiostal hematoma. The patient underwent a surgical intervention to drain the hematoma when an orbital mass was found. The microscopic, immunologic, and genetic features of the tumor and the myelogram were in favor of LAM2, and the patient was started with chemotherapy with a favorable evolution within 18 months follow-up.

**Conclusion:**

Orbital myeloid sarcoma usually exhibits clinical and radiological features that can be easily misleading, especially if it happens *de novo* or as the first manifestation of acute myeloid leukemia. Only a few isolated cases have reported and proposed trauma as a trigger event of the onset of this type of tumor proliferation, but further investigations and evidence are needed to support this hypothesis.

## Introduction

Myeloid sarcoma (MS), also known as granulocytic sarcoma (GS) or chloroma, is a solid tumor lesion that consists of cells of the myeloid lineage in varying stages of maturation, occurring at an extramedullary site [1]. MS can represent a complication of different myelodysplastic syndromes. It is commonly associated with acute myeloid leukemia (AML) and is hence known as extramedullary manifestation of AML. MS occurs in approximately 4–5% of children with acute myeloid leukemia and may develop before, during, or after the occurrence of AML [2, 3]. Only a few isolated cases of pediatric orbital MS have been reported, and they are often associated with a high misdiagnosis rate.

Orbital MS usually exhibits clinical and radiological features that can be easily misleading, especially if it happens *de novo* or as the first manifestation of AML. Trauma as a cause of MS is unusual, and it is a controversial predisposing factor in the initiation of tumor proliferation. Hence, patient’s history, clinical findings, and conventional radiological investigations may not be sufficient to list MS as a possible diagnosis in front of orbital blunt trauma. This case is reported here for its rarity and its unusual misleading presentation that could delay diagnosis and treatment.

## Case report

**Fig. 1:**
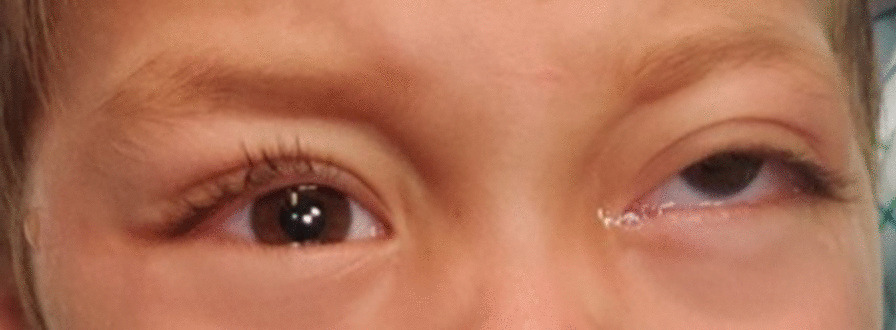
Photograph of the patient showing the upward displacement of the left globe

**Fig. 2:**
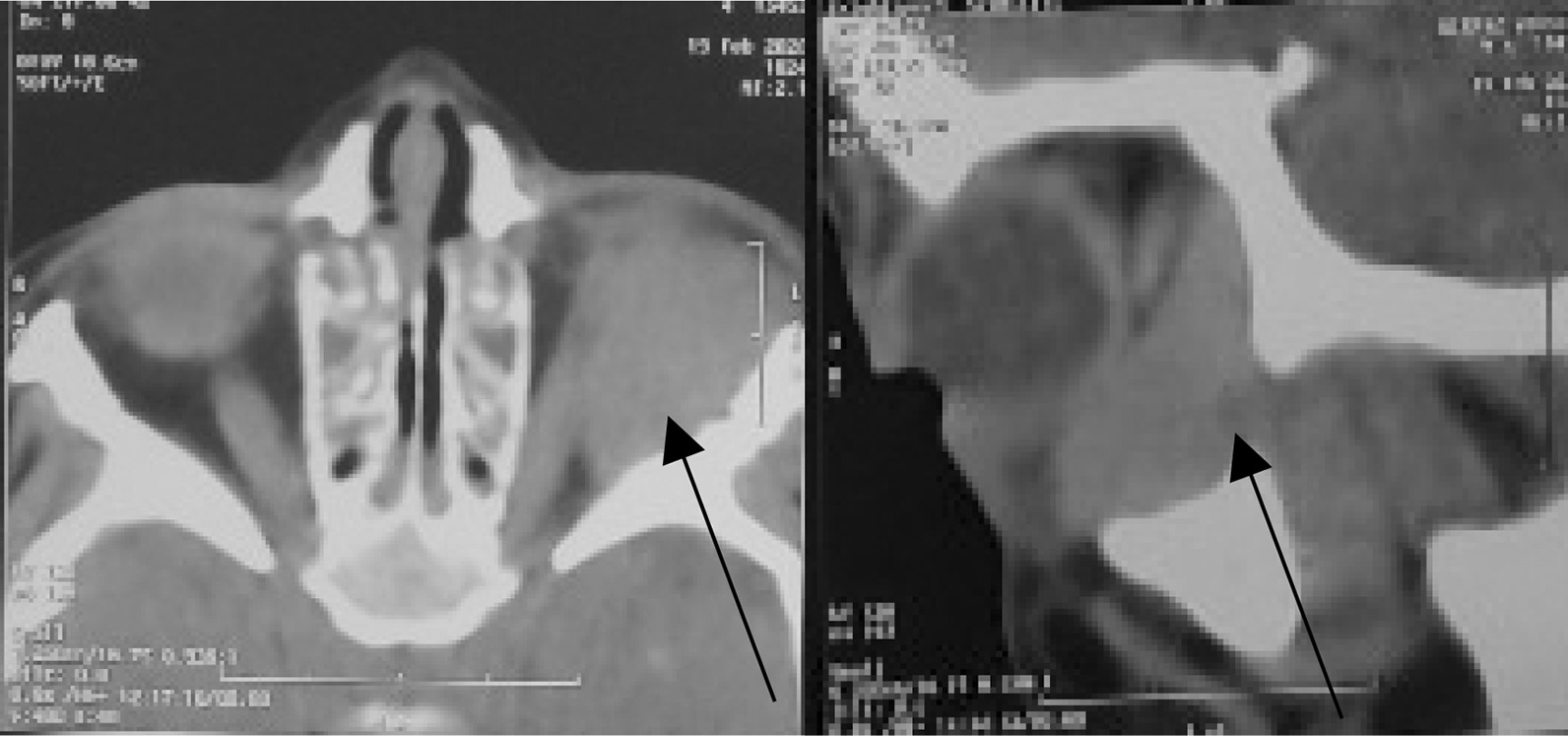
Craniofacial computed tomography showing a spontaneously hyperdense, oval-shaped, extraconal mass repressing the inferior rectus muscle of the left orbit (arrows)

**Fig. 3:**
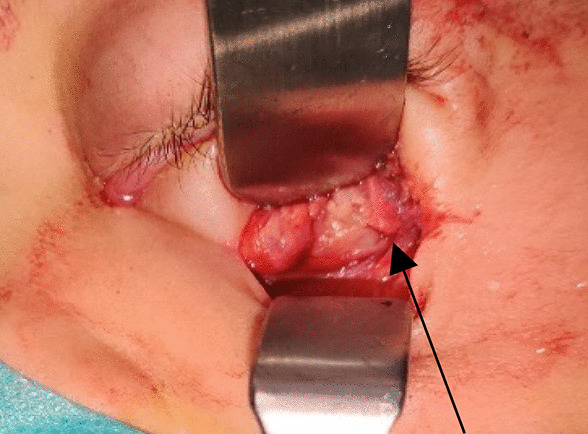
A photograph of the intraorbital tumor above the orbital floor (arrow)

A 3-year-old Caucasian male patient presented to us with a swelling of 2 weeks duration in the left eye. The onset was gradual and was preceded by blunt eye trauma in a traffic accident. The patient was initially admitted to another hospital, where the initial examination revealed a left periorbital ecchymosis with subconjunctival hemorrhage and proptosis without any vision loss. After 2 weeks of surveillance, the patient presented a persistent proptosis for which he was addressed to our structure. The admission examination found a slight proptosis of the left eye with a tender swelling palpable at the inferior orbital region that was pushing the globe upwards (Fig. 1). Movements of the globe as well as pupillary function were normal. No abnormalities were found in the examination of the anterior and posterior segments. His intraocular pressure in both eyes was in the average range. Neurological examination found no signs of intracranial hypertension, of sensory or motor deficiency. The rest of the physical examination found no signs of distant lesions such as osteoarticular pain, hepatomegaly, splenomegaly, enlarged lymph nodes, or dullness of the flanks. No notable history of hematopathy or weight loss were presented, and the patient’s parents are not related. A craniofacial computed tomography (CT) was performed showing a spontaneously hyperdense, oval-shaped, extraconal mass measuring 29 × 19 mm without significant enhancement after contrast injection. This mass repressed the inferior and external rectus muscles and was not associated with any bone abnormalities (Fig. 2). In front of these clinical and radiological findings, the first diagnosis proposed was an inferolateral subperiostal hematoma. An inferior mediopalpebral incision was performed to drain the hematoma when a pale yellowish tumor was found against the orbital floor (Fig. 3). An excision of the mass was done. Microscopy showed the features of a round-cell tumor infiltrating the fibrocollagenous tissue. On immunohistochemistry, cells expressed positivity for anti-Myeloperoxydase (anti-MPO) and CD-45, and were negative for S-100 and myogenin. Complete blood count revealed: hemoglobin (Hb) 11.9 g/dl, total leucocyte count (TLC) 19540/mm^3^ with 20% blasts. Liver and renal functions were within normal limits [alanine aminotransferase (ALT) 23 UI/L, aspartate aminotransferase (AST) 17 UI/L, total bilirubin 11.7 mg/L, urea 0.27 g/L, creatinine 6.8 mg/L]. Human immunodeficiency virus (HIV), hepatitis, and syphilis serology was negative. Blood culture and cytobacteriological examination of urine revealed no pathogens. White blood cells in the cerebrospinal fluid were < 5 elements/mm^3^. Bone marrow aspirate smears revealed the presence of 24% blasts, and the diagnosis of orbital myeloid sarcoma with acute myeloid leukemia was made. The patient was t (8;21) (q22;q22) positive, and the subtype was AML M2. Thus, the patient was started on induction therapy, with intravenous cytarabine (Ara-C: 15 mg/12 hours the first, second, and third day of chemotherapy) and daunorubicin (10 mg/day, the third and fourth day), followed by consolidation with both molecules plus intrathecal administration of methotrexate, Ara-C for two doses. On the 18th month follow-up, the patient was disease free.

## Discussion

Our paper is a case report of an exceptional pediatric orbital myeloid sarcoma appearing after a blunt trauma and initially misdiagnosed as a subperiostal hematoma, which has never been reported in the literature. The first description of chloroma was reported in 1811 by Allen Burns, who described a green tumor involving the orbit. The green color is due to the exposure of the enzyme myeloperoxidase to ultraviolet light [4–6]. Rappaport renamed it granulocytic sarcoma in 1966, because up to 30% of granulocytic sarcomas do not display a green color [4, 6]. The term extramedullary myeloid tumor (EMMT) was proposed by Davey *et al*. [7], and it was not until 2002 when the name “myeloid sarcoma” was declared by the World Health Organization (WHO) [8].

Myeloid sarcoma may occur as a separate entity, then called primary MS or *de novo* [2, 9], or secondarily associated with AML, chronic myeloid leukemia (CML), myelodysplastic syndrome (MDS), polycythemia vera (PV), myelofibrosis, and essential thrombocytosis [2]. MS is a rare condition occurring in only 2.5–9.1% of patients with AML [4, 10]. Of these cases, 15–35% are concomitant with AML, 25% precede AML, and up to 50% of the cases occur after the diagnosis of AML. It might as well be the initial manifestation of relapse [1]. It affects mostly children, since 60% of MS patients are younger than 15 years old [11], with a median age at diagnosis of 2.8 years (range, 1 month to 18 years) [9]. The most common sites of MS include skin, bone, gum, soft tissues, orbit, lymph nodes, mediastinum, peritoneum, small intestine, paranasal sinuses, epidural sites, lung, heart, bladder, uterus, and ovaries [2, 9, 10, 12, 13]. The orbit appears to be a favored site for MS in the pediatric population [9, 14].

Proptosis is the most common presenting clinical sign of orbital MS. Some of the various other presentations described include lacrimal gland swelling, chemosis, epiphora, eyelid tumor, iris tumor, uveitis, conjunctival mass, ophthalmoplegia, vision loss, optic neuropathy, exotropia, and scleral mass [4–6, 15, 16]. It has been reported that orbital MS on CT appears as a well-defined mass, which may arise intraconally or extraconally. It molds to the bone and contiguous structures, including the sclera and the orbital bones. The mass is usually homogeneous, isodense, or hyperdense to brain tissue, with homogeneous enhancement after contrast media injection. However, in some cases, it can exhibit some nonenhancing areas corresponding to the inner necrotic areas, which is considered as a sign of rapid growth [4, 11].

In the absence of a known history of myelodysplastic syndromes, MS can be easily misdiagnosed as a number of other neoplastic diseases depending on the site of involvement. the rate of misdiagnosis has been reported to be in the range of 25–47% [12]. Orbital MS is often considered a diagnostic dilemma, and the differential diagnosis includes malignant lymphoproliferative disorder, primitive neuroectodermal tumor, Ewing’s sarcoma, rhabdomyosarcoma, neuroblastoma, small round-cell tumors, retinoblastoma (especially in pediatric patients), and metastasis [1, 2, 8]. Other more challenging mimickers to be considered include vascular lesion, orbital cellulitis, and hematoma [4, 5, 11, 14]. In our case, due to the clinical and radiological features and to the patient’s history of blunt trauma, subperiostal hematoma (SPH) was the diagnosis to evoke. SPH is due to relatively loose attachment between the periosteum and the orbital bones, except at the suture, thus creating a potential space for the hematomas to develop. With age, this attachment becomes firmer, which accounts for the decreased incidence in adults [17, 18]. Traumatic SPH occurs either immediately or within days following orbital trauma. The characteristic findings include proptosis, displacement of the globe, chemosis, motility impairment, and varying degrees of visual impairment [18]. The hematoma is recognizable by its morphology reminiscent of the lentiform shape of an intracranial epidural hematoma, with a broad base, displacement of the orbital content, and optic nerve stretching. As with hematomas in other locations, SPH is spontaneously hyperdense with respect to skeletal muscles and does not enhance, and is therefore hypodense relative to the extraocular muscles on contrast-enhanced CT [17–19]. The differential diagnosis includes neoplasms and inflammation [19]

MS is thought to occur in bone marrow and then spread via Haversian canals to penetrate periosteum and then form a soft-tissue mass. This would account for the typical location near bony structures, which is the case of orbits. The exact mechanism of extramedullary spread is not fully understood. However, some studies found that deregulation of core-binding factor transcription factors, which are involved in cellular adhesion and recognition, may be part of the pathogenesis of MS. The blast neural adhesion molecule (CD56) may have a role in the pathogenesis as well; it has been supported by the fact that a high incidence of MS has been associated with CD56 blast expression, common with t (8; 21). Chemokine receptor–ligand interaction that orchestrates the migration of cells to peripheral tissues has also been incriminated, since AML blasts were found to express some chemokine receptors not seen in blasts of bone marrow and peripheral blood [8, 14, 20].

Blunt trauma can cause local tissue degeneration, and necrosis, and finally, result in cell atypia during the healing process. Only a few isolated cases had history of orbital trauma preceding the onset of MS and proposed trauma to be a possible trigger event for the onset of this type of malignancy [5, 15, 21]; however, it remains a controversial risk factor in the onset of MS, and further evidence is needed to verify this hypothesis. Most of the reported cases that had a hematologic tumor after trauma usually developed to non-Hodgkin’s lymphoma [15].

White blood cell (WBC) count at diagnosis, French–American–British (FAB) immunologic subtypes, and cytogenetics are the main important prognostic factors and, thus, must be performed for all patients [3, 22]. Studies have demonstrated that monosomal karyotypes are independent risk factors for poor prognosis, that MS patients with chromosome 8 abnormalities had a worse prognosis, and that intensive chemotherapies were needed in these groups [23, 24]. Overall, an extramedullary leukemic infiltration is a bad prognostic sign, because it might act as a reservoir for proliferation of leukemic cells and an eventual relapse [6, 25]. For this reason, primary myeloid sarcoma should be treated as AML even in the absence of clinically detectable AML. The treatment modalities include induction and consolidation therapy with regiments containing citarabin similar to those with AML [1, 12, 15]. Radiotherapy and debulking surgery could be used in conjunction with systemic therapies, primarily in patients who need rapid symptomatic relief. In the case of orbital involvement, radiation could be used to obtain relief from compressive symptoms and to reduce visual deterioration, and it may also be useful to avoid enucleation [2, 10, 12]. Bone marrow transplantation or hematopoietic stem cell transplantation (HCST) and targeted therapy based on agents such as FLT3 inhibitors, farnesyltransferase inhibitors, histone deacetylase inhibitors, and DNA methyltransferase inhibitors could also be considered as therapeutic options for MS patients.

## Conclusion

Orbital myeloid sarcoma is a rare form of extramedullary leukemic infiltration. Blunt trauma is rarely associated with such tumor proliferation and needs more investigation before being accepted as an actual risk factor, though it still deserves more attention for early diagnosis and timely and proper treatment. Immunohistochemical analysis is vital for the early diagnosis of this kind of disease, which is usually misdiagnosed. Systemic chemotherapy is more effective than radiotherapy or surgery alone, even in the absence of bone marrow infiltration.

## Data Availability

Data and materials supporting this work are available for review by the Editor-in-Chief of this journal on request.
